# Oral diet management for carcinoma at the base of tongue with radiotherapy and chemotherapy associated dysphagia: a case report

**DOI:** 10.3389/fnut.2023.1239911

**Published:** 2023-10-05

**Authors:** Zhen Ding, Lingmei Zhou, Kemei Jin, Runjinxing Wu, Yihua Gui

**Affiliations:** ^1^Clinical Nutrition Department, Ningbo Medical Center Li Huili Hospital, Ningbo, China; ^2^Department of Otolaryngology, Head and Neck Surgery, Ningbo Medical Center Li Huili Hospital, Ningbo, China

**Keywords:** tongue cancer, dysphagia, thickener, nutritional management, case report

## Abstract

**Introduction:**

Tongue cancer is one of the common malignancy of the head and neck, and directly impacts chewing, swallowing, and other eating activities. Based on the evidence-based guidelines and clinical management, this paper presents nutrition management experience of a patient with tongue cancer who had a dysphagia and feeding reflux while undergoing radiotherapy and chemotherapy.

**Methods:**

Nutritional risk screening and comprehensive nutritional assessment were performed based on the patient’s medical history, and personalized nutritional programs were developed under the guidance of the clinical pharmaceutical consensus of parenteral nutrition and nutritional treatment guidelines for patients with tumors during radiotherapy. For the management of oral feeding, the patient’s swallowing function was evaluated to manage oral feeding. Thickening powders were used to improve the consistency of the patient’s food, which successfully achieved oral feeding of the patient.

**Results:**

The patient finally ate five meals a day by mouth, and energy requirements were met using industrialized nutritional supplements, and homogenized food was added in between the meals. The energy provided by enteral nutrition can reached approximately 60–75%. The patient’s weight and albumin levels had increased significantly at the time of discharge.

**Discussion:**

The nutritional management of patients with dysphagia should be jointly managed by clinicians, nurses, nutritionists, and family members to effectively improve the quality of life (QOL) and nutritional status of patients. To ensure adequate nutritional supply, appropriate swallowing training may delay the deterioration of the chewing function and improve the eating experience of such patients.

## 1. Introduction

Patients with head and neck cancer (HNC) are the most vulnerable in terms of cancer-related malnutrition before, during, and after cancer treatment (1, 2). Between 44 and 50% of the patients with HNC present with dysphagia, either as a disease symptom or following chemotherapy (3), and this aggravates existing malnutrition. However, for patients with swallowing dysfunction, the nutrition is often implemented through nasogastric tube or percutaneous gastrostomy, which is prone to cause complications, such as nasopharyngeal irritation, infection, and diarrhea. It is difficult to tolerate for a prolonged time and can cause a loss of pleasure for eating (4, 5). Swallowing training and nutritional intervention for patients with swallowing dysfunction attracts increasing attention (6). Thus, we reviewed the oral diet management for the base of tongue carcinoma-associated dysphagia.

## 2. Case description

On November 4, 2019, a 57-year-old inarticulate woman with foreign body sensation at the base of tongue persisting for over a year, was admitted to the radiotherapy department of Ningbo medical center Li Huili Hospital, in China. In the past few months, CT was performed, and the epithelial malignant tumor was diagnosed. During the past year, the patient’s tumor was in the advanced stage and had not been actively treated. The CT showed the pharynx and larynx malignancy with multiple bone destruction and enlarged left cervical lymph nodes. According to the American Joint Committee on Cancer (AJCC) staging system, the patient was classified as being at stage IV. Histologically, most tumor cells were epidermal and basal cell-like, with a little cell differentiation, inclined towards medium grade mucoepidermoid carcinoma. Chemotherapy (carboplatin 300 mg d1 + docetaxel 80 mg d1) had been previously administered on September 17 and August 8, 2019, in Ningbo Medical Center at the Li Huili Hospital.

### 2.1. Clinical treatment after admission

After this hospitalization, enhanced CT of the oropharynx showed irregular patchy T1 and slightly longer T2 signal shadow on the left side of the tongue root area, with irregular shape and unclear boundary. The left pyriform recess was narrow and occluded, with mixed shape, and the range was approximately 32.9 × 37.7 × 22.9 mm. An enlarged lymph node could be seen in the deep part of the left neck. Imaging diagnosis was left tongue base cancer with left neck lymph node metastasis. After admission, the patient received radiotherapy 5 times a week. The regimen was as follows: 95%PTV1 DT6000cGy/30f, 95%PTV2 DT5040cGy/28f, 95%PGTVnx DT6420cGY/30 f. Since the patient could not tolerate simultaneous chemotherapy (docetaxel 80 mg d1), so vascular targeted therapy was performed instead (Recombinant Human Endostatin Injection 15 mg qd d1-d14).

### 2.2. Clinical nutrition support after admission

**Figure 1:**
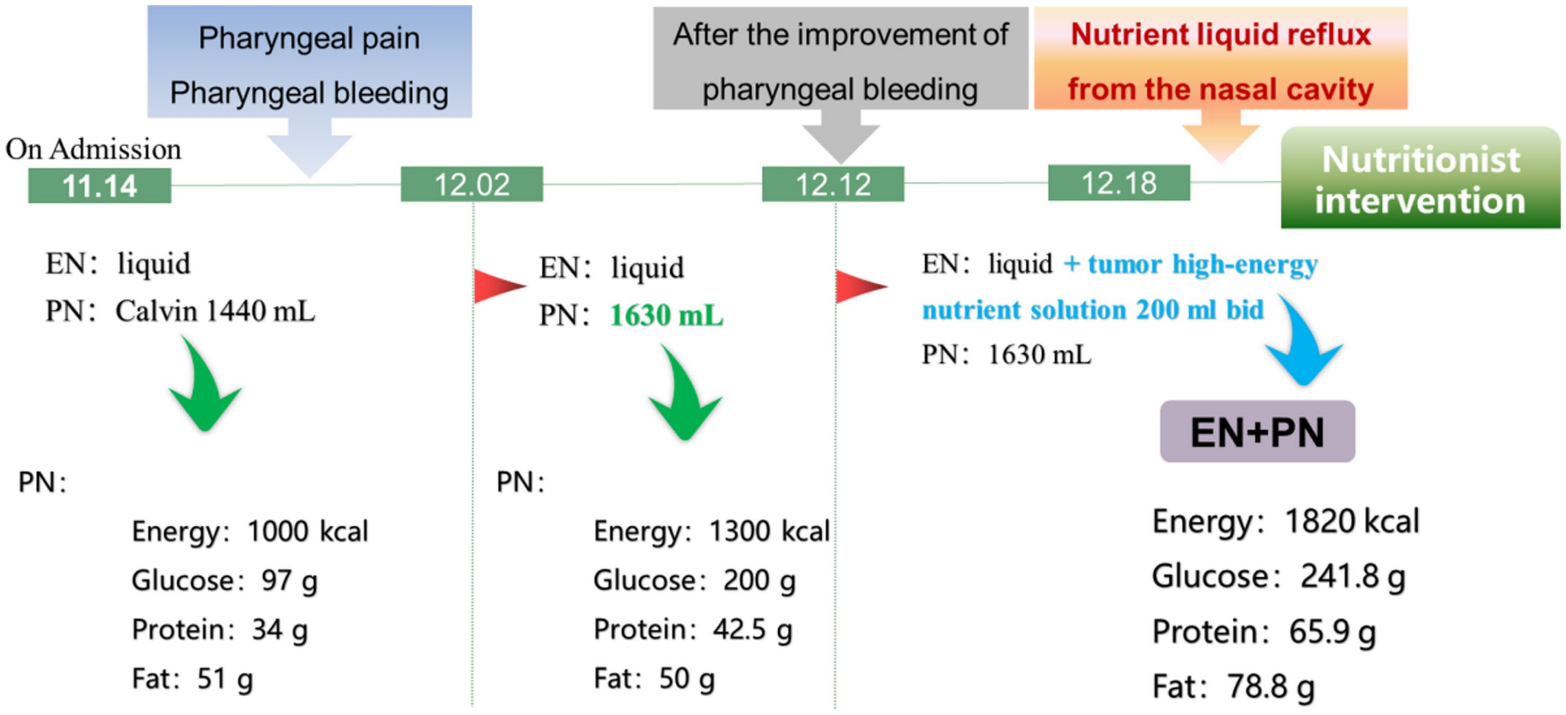
Clinical nutrition support history before nutritionist intervention.

Due to the limitations of mouth opening, the patient was only able to drink some liquids, which could not meet daily nutritional requirement. Supplemental parenteral nutrition was added [1,440 mL fat emulsion, amino acids and glucose (11%) injection]. Radiotherapy was started on the 4th day of admission, five times a week. After the 6th times, the patient developed radiation pharyngitis and pharyngeal hemorrhage due to vascular targeted therapy, which treated with reduce swelling, prevent infection, suspension of vascular targeted therapy and analgesia. Besides, parenteral nutrition was enhanced. After the radiation pharyngitis and pharyngeal hemorrhage improved, a tumor high-energy nutrient solution (Specification: 200 mL/bottle, energy density: 1.3 kcal/mL, energy source: 32% carbohydrate, 50% fat, 18% protein) was added orally. However, on the 6th day of increased nutritional support, the patient experienced nutrient liquid reflux from the nasal cavity and a nutritionist had to be consulted. The detailed process is illustrated in Figure 1.

### 2.3. Comprehensive nutrition assessment

The physical parameters for the patient were as follows: height: 155 cm, weight: 36.6 kg, BMI: 15.2 kg/m^2^. Before admission, the patient had an unspecified weight loss. In the past 1 month, no significant weight loss was observed. The patient’s food intake gradually decreased, and she undergone a fluid diet. Based on these, the total score of the nutritional risk screening (NRS2002) was 4, which indicated that the patient was at nutritional risk. The subjective global assessment scale (PG-SGA) score for tumor patients was 14, indicating that the patient was severely malnourished. The albumin value on the day of the nutritional consultation was 32.3 g/L (normal value: 40–55 g/L).

In accordance with the Chinese Society of Clinical Oncology (CSCO) 2019 Edition Guidelines for Nutritional Treatment of Malignant Tumor Patients (7), we calculate the target energy and protein levels of 1,250–1,500 kcal/d and 75–100 g/d, respectively, based on the patient’s targeted daily energy of 25–30 kcal/kg and daily protein of 1.5–2.0 g /kg (ideal weight). Next, implementing effective oral feeding was the second goal.

### 2.4. Nutrition support process

#### 2.4.1. Enhancement of parenteral nutrition

For cancer patients, the parenteral nutritional formula should be adjusted and the ratio of non-protein calories (NPC) to nitrogen should be noted. Patients whose tumors cannot be completely resected surgically can increase their fat intake to adapt to changes in their body metabolism (8). Therefore, 8.5% of the compound amino acid injection (18AA-II) and 20% of the medium and long chain fat emulsion injection (MCT/LCT) were increased to 750 mL and 375 mL, respectively. The adjusted parenteral nutrition provided 1,610 kcal energy (32 kcal/kg), 63.75 g protein (1.3 g/kg), and NPC: Nitrogen = 133:1.

#### 2.4.2. Evaluation of swallowing function

The patient showed deglutition-specific symptoms such as decreased food intake, choking while drinking, enteral nutrient fluid reflux from the nasal cavity, and a hoarse voice. Then the volume viscosity swallowing test (V-VST) was used to evaluate swallowing ability (9). Thickening powders were added to three cups containing 140 mL water to form liquids with the consistency of syrup (6.4 g), honey (9.6 g), and pudding (12.8 g). Under the guidance of nurse and nutritionist, the patient was allowed to drink water of different consistencies with monitoring blood oxygen levels. As result, the patient experienced cough, voice changes, and other symptoms when swallowing 10 mL syrup-consistency liquid. When swallowing 10 mL and 20 mL of honey-consistency liquid, the aforementioned symptoms did not appear. Therefore, 20 mL of honey-consistency liquid was deemed appropriate for the patient.

#### 2.4.3. Adjustment of texture and consistency

For patients with dysphagia, nutritional management refers to the improvement of food quality and traits including changes in the structure or viscosity of food. Nutritional management refers to the improvement of food quality and traits including changes in the structure or viscosity of food. Viscosity is the only rheological property affected by dietary modifications for the management of dysphagia (10, 11). Based on the V-VST results, the patient’s food intake was regulated to maintain consistency.

In the first stage of oral feeding (day 1 to 3 of the nutrition department intervention), considering that patients had been on liquid food for a long time, the initial food was 140 mL rice soup (five times a day) with 9.6 g of added thickening powder. On the second day, the rice soup was reduced to three times a day (tid) and 140 mL balanced nutrient solution fortified with 10 g whey protein powder (high protein nutrient solution), twice a day (bid). The high-protein nutrient solution provided 446 kcal energy and 30 g protein. On the third day, the patient was administered 140 mL rice soup once a day (qd), 140 mL high protein nutrient solution (bid), and 100 mL tumor type nutrient solution (bid). Enteral nutrition provided 706 kcal energy and 42 g protein. During this stage, the energy provided exceeded 50% of the target energy level, and the patient did not choke or cough after eating. The menu for the first 3 days is listed in Table 1. During the second stage of oral feeding (day 4 to 10 of the nutrition department intervention), we attempted to adjust the consistency of natural foods to enrich the patient’s taste. On the initial 3 days, natural food was added at lunch so that the nutritionist could follow up after eating. The patient consumed five meals per day, which mainly consisted of enteral nutrition liquid, and natural food homogenates were added in the morning and afternoon. The weekly recipes are listed in Table 2. The amount of high-protein nutrient solution was 140 mL, and the amount of tumor-type nutrient solution was 100 mL. Natural food homogenate is a honey consistency created by crushing cooked food to a liquid state with a mixer and adding thickening powders.

**Table 1:** Diet management during the first stage (day 1 to 3 of nutritionist intervention).

Meals	Day 1	Day 2	Day 3
Breakfast	Rice soup	Rice soup	Rice soup
Morning dim sum	Rice soup	High protein nutrient solution	High protein nutrient solution
Lunch	Rice soup	Rice soup	Tumor type nutrient solution
Afternoon snack	Rice soup	High protein nutrient solution	High protein nutrient solution
Dinner	Rice soup	Rice soup	Tumor type nutrient solution

**Table 2:** Diet arrangement during the second stage (day 4 to 10 of nutritionist intervention).

Meals	Day 4	Day 5	Day 6	Day 7	Day 8	Day 9	Day 10
Breakfast	High protein nutrient solution	High protein nutrient solution	High protein nutrient solution	Rice paste with red dates and millet	High protein nutrient solution	Rice paste with oatmeal	High protein nutrient solution
Morning dim sum	Tumor type nutrient solution	Tumor type nutrient solution	Tumor type nutrient solution	High protein nutrient solution	Tumor type nutrient solution	Tumor type nutrient solution	Rice paste with pumpkin and egg
Lunch	Rice paste with pumpkin and egg	Rice paste with spinach and pig liver	Rice paste with sweet potato	Rice congee and homogenate with tomatoes and fish	Rice paste with meat and yum	Rice congee and homogenate with cauliflower and shrimp	High protein nutrient solution
Afternoon snack	Tumor type nutrient solution	High protein nutrient solution	High protein nutrient solution	High protein nutrient solution	High protein nutrient solution	High protein nutrient solution	Rice congee and homogenate with meat and carrot
Dinner	High protein nutrient solution	High protein nutrient solution	Tumor type nutrient solution	High protein nutrient solution	Tumor type nutrient solution	Tumor type nutrient solution	High protein nutrient solution

#### 2.4.4. Nutritional outcome

After adjusting the nutrition scheme, the patient received enteral and parenteral nutrition support, which provided 2,530 kcal/d energy and 97.5 g/d protein. Therefore, the nutritional supply was sufficient. The energy provided by oral feeding reached more than 60% of the target energy, reaching the standard of breaking away from parenteral nutrition support and laying the foundation for nutritional support of patients after discharge.

**Figure 2:**
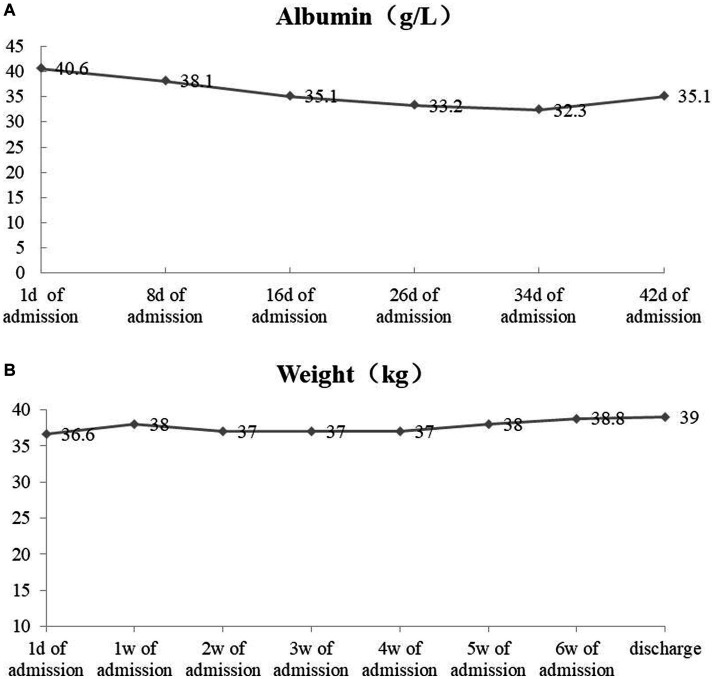
Albumin level **(A)** and weight **(B)** monitoring.

During nutritional support, weight and albumin levels increased, as shown in Figure 2. Enhanced CT of the oropharynx showed that the left tongue root tumor was significantly smaller than that in September (29.5 × 41.6 mm).

## 3. Discussion

For HNC patients, post-treatment complications are manifested by poor swallowing function and physiology (12). Generally, the causes are roughly divided into two categories: neuromuscular dysfunction and structural abnormality (organic) (13). Due to the invasion and compression of the tumor and the decreased elasticity of soft tissue, the feeding channel is obstructed. Otherwise, it is caused by the throat muscle paralysis caused by tumor invasion of nerves or tumor treatment. In clinical practice, to prevent malnutrition in such patients, enteral nutrition through nasal gastrostomy and jejunostomy feeding or parenteral nutrition through intravenous feeding is adopted. However, patients undergoing a long-term gastrostomy may have difficulty swallowing and rely on tube feeding (14). Therefore, these patients should be encouraged to maintain proper oral feeding.

However, for patients with dysphagia, it is necessary to determine two deglutition-defining characteristics: efficacy and safety during oral consumption. To assess both characteristics of deglutition, two groups of diagnostic methods are available: (a) clinical screening methods such as deglutition-specific medical history and clinical examination, and (b) the exploration of deglutition using specific complementary studies such as fiberoptic endoscopic evaluation of swallowing (FEES) or video fluoroscopy (VFS) (15). Clinical screening should be low risk, quick, low cost, and can include the Eating Assessment Tool (EAT-10), standardized bedside swallow assessment (SBSA), and the Toronto Bedside Swallowing Screening Test (TOR-BSST) (16–18). In this case, the V-VST was used, which is a safe, quick, and accurate clinical method with 88.2% sensitivity for impaired safety, 100% sensitivity for aspiration, and up to 88.4% sensitivity for impaired efficacy of swallows. A series of 5–20 mL nectar, liquid, and pudding boluses sequentially administered in a progression of increasing difficulty. Cough, fall in oxygen saturation 3%, and changes in quality of voice were considered the clinical signs of impaired safety, whereas piecemeal deglutition and ropharyngeal residue were treated as signs of impaired efficacy (19). After swallowing function screening, the physical properties of food affecting the swallowing function, would be determined (20). Dilute liquids are most likely to reduce aspiration; therefore, food texture and consistency modifications are common practices in nutritional management (9, 21). Karagiannis et al. (22) reported significantly lower incidence of aspiration pneumonia in a group taking viscous liquids than that in a group taking water. However, patients with dysphagia are not recommended to consume rice paste, sesame paste, or other natural food in paste form that are not processed using thickened conditioners. These foods can remain in the mouth and pharynx, causing aspiration and increasing the risk of pneumonia *via* inhalation. The use of food thickeners is the basis for management of dysphagia and can improve the swallowing safety and prevent aspiration (23). Functional foods for use in dysphagia have corresponding texture characteristics, such as loosening difficulty and proper viscosity, which help them pass through the mouth and pharynx smoothly and reduce the risk of aspiration (24).

After clinical symptom screening and V-VST assessment of our patient, food with a honey consistency was deemed most appropriate. Finally, the patient ate up to five meals per day orally, while industrialized nutritional preparations provided main nutritional support. Natural food homogenates were used as snacks between meals. The energy provided by enteral nutrition reached 60–75%, which met the requirements for stopping parenteral nutrition support. After discharge, parenteral nutrition cannot be implemented. However, the patient explicitly believed that she could tolerate current enteral nutrition well and master the use of food thickeners. The combination of industrial nutritional preparations and natural food homogenization not only meets the nutritional requirements of patients but also enhances their eating experience, that would improve quality of life.

This case had several limitations. During hospitalization, the patient did not undergo any fiberoptic endoscopic evaluation of swallowing and systematic swallowing rehabilitation. Our management focused on increased oral consumption through food texture and consistency modifications. Otherwise, the patient was discharged unexpectedly, so that, we could not gradually reduce parenteral nutrition support as it was planned. Additionally, the patient was not followed-up regularly in the nutrition clinic; therefore, it was impossible to monitor the oral food and nutritional status of the patient after discharge. Thus, we should strengthen nutrition supervision after discharge and promptly assess whether oral feeding continues to meet the nutritional requirement. If this fails, tube feeding nutritional support should be added in a timely manner.

Dietary nutrition management in patients with dysphagia has obvious particularities compared with other types of disease. Professional clinical staff are required to assess the swallowing ability, and nutritionists should formulate personalized nutritional prescriptions. Multidisciplinary cooperation is necessary to provide sufficient nutrition for patients, reduce tube feeding dependency, achieve early oral feeding, delay the degradation of chewing function, improve nutritional status, make patients eat and enjoy food, improve quality of life, and improve clinical outcomes. The multidisciplinary cooperation needs to extend to the outpatient clinic, so that patients’ nutritional status during the recovery period after discharge can be monitored and adjusted.

## Data availability statement

The raw data supporting the conclusions of this article will be made available by the authors, without undue reservation.

## Ethics statement

The studies involving humans were approved by Ethics committee of Ningbo Medical Center Li Huili Hospital (KYSB2020YJ044-01). The studies were conducted in accordance with the local legislation and institutional requirements. The participants provided their written informed consent to participate in this study. Written informed consent was obtained from the individual(s) for the publication of any potentially identifiable images or data included in this article.

## Author contributions

ZD contributed to personalized nutrition plan and writing and editing of the manuscript. KJ, LZ, and RW contributed to nutritional management. YG contributed to patient care. All authors contributed to the article and approved the submitted version.
